# An Unusual Cause of Obstructive Jaundice and Acute Pancreatitis: Visceral Artery Pseudoaneurysm

**DOI:** 10.7759/cureus.14307

**Published:** 2021-04-05

**Authors:** Akira Saito, Nabil Fayad

**Affiliations:** 1 Gastroenterology and Hepatology, Indiana University, Indianapolis, USA

**Keywords:** acute pancreatitis, visceral artery pseudoaneurysm, biliary obstruction

## Abstract

Visceral artery pseudoaneurysm (VAPA) is an uncommon vascular disorder with a tendency to present with nonspecific signs and abdominal symptoms. This case describes a patient with severe atherosclerosis who developed multiple VAPAs including a hepatic artery pseudoaneurysm bleeding into a large hematoma, which resulted in obstructive jaundice and acute pancreatitis. Prompt diagnosis of VAPA is important due to the high risk of vessel wall perforation with associated increased mortality rate. Biliary obstruction with acute pancreatitis is not a well-described presentation for VAPAs.

## Introduction

Visceral artery pseudoaneurysm (VAPA) is a rare vascular disorder that can present with a variety of symptoms depending on the affected vessel and lesion size [1]. Risk factors for the development of VAPAs include atherosclerosis, trauma, iatrogenic injury, infection, inflammatory states, vasculitis, and connective tissue disorders. Early recognition of this rare disorder with cross-sectional imaging is important as there is a high risk of perforation of the affected blood vessel along with a high risk of mortality due to hemorrhage [2,3]. Here, we report an unusual case of a patient with multiple VAPAs whose initial clinical presentation included jaundice, acute pancreatitis, and acute kidney injury. 

## Case presentation

A 70-year-old male with a history of atherosclerosis, coronary artery disease, congestive heart failure, atrial fibrillation on anticoagulation, femoral artery aneurysm repair, and cholecystectomy 20 years ago, presented to the emergency department with one week of abdominal pain, nausea, and vomiting. On presentation, he was noted to be hemodynamically stable, jaundiced, and epigastric abdominal tenderness. Laboratory studies revealed a hemoglobin of 10.6 g/dL, creatinine of 3.51 mg/dL (patient baseline of 1.0 mg/dL), total bilirubin 9.5 mg/dL, alkaline phosphatase 953 units/L, aspartate aminotransferase (AST) 66 units/L, alanine aminotransferase (ALT) 58 units/L, and lipase 1529 units/L (upper limit of normal is 60 units/L).

**Figure 1:**
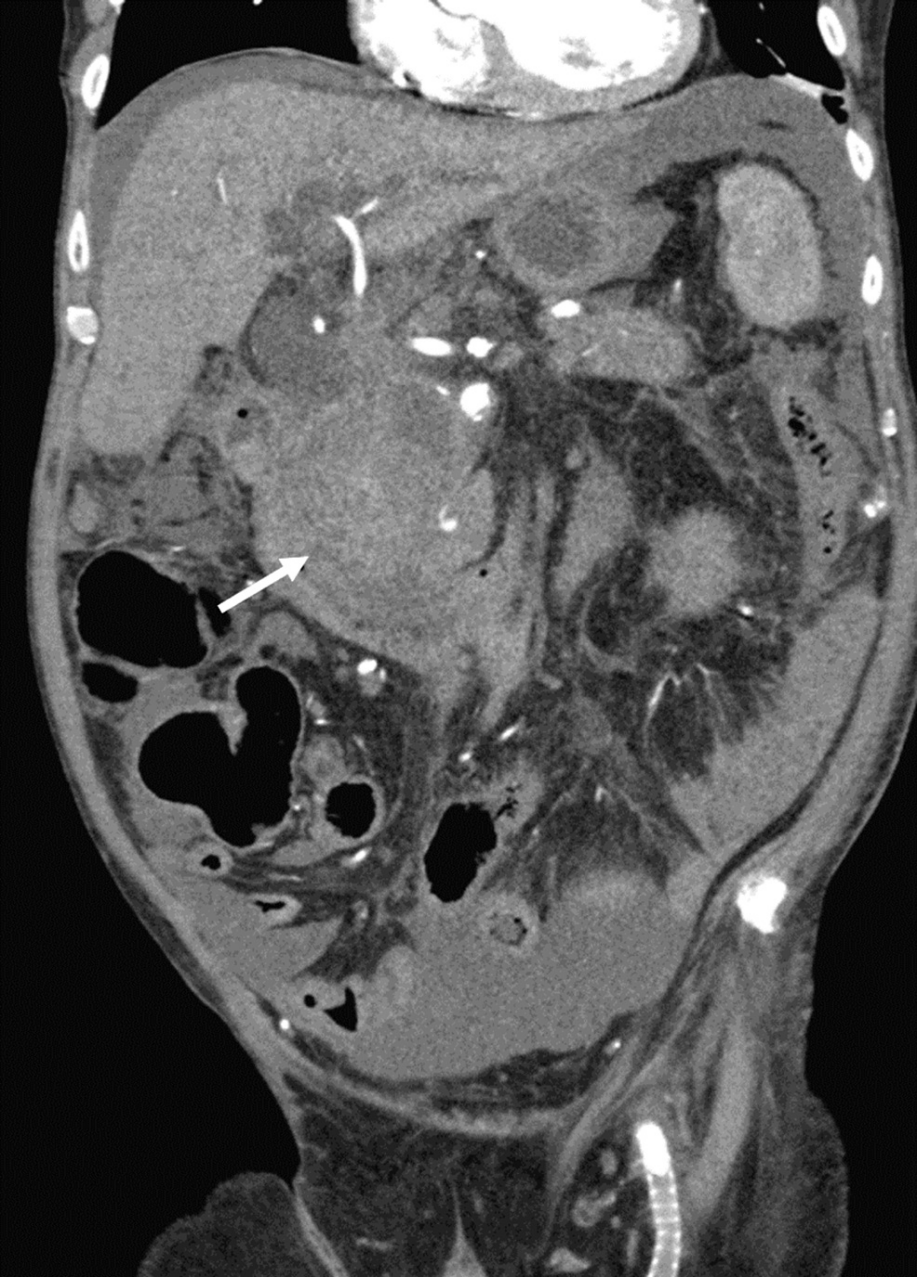
Non-contrasted CT scan of the abdomen and pelvis (coronal view). The white arrow points at a large, abnormal heterogeneous structure that was found at the level of the head of the pancreas. Due to the mass effect of this structure, there is common bile duct compression and upstream dilatation of the biliary system. Intrahepatic ductal dilatation can be appreciated.

A CT abdomen and pelvis was performed without IV contrast due to acute kidney injury (Figure 1). This revealed a large heterogeneous and hyperattenuating structure at the level of the head of the pancreas close to the superior mesenteric artery (SMA) with associated mass effect on the duodenum and obstruction of the common bile duct. Marked dilatation of the intra and extra-hepatic biliary tree was also noted. These findings were not seen on CT abdomen with contrast four months prior. 

**Figure 2:**
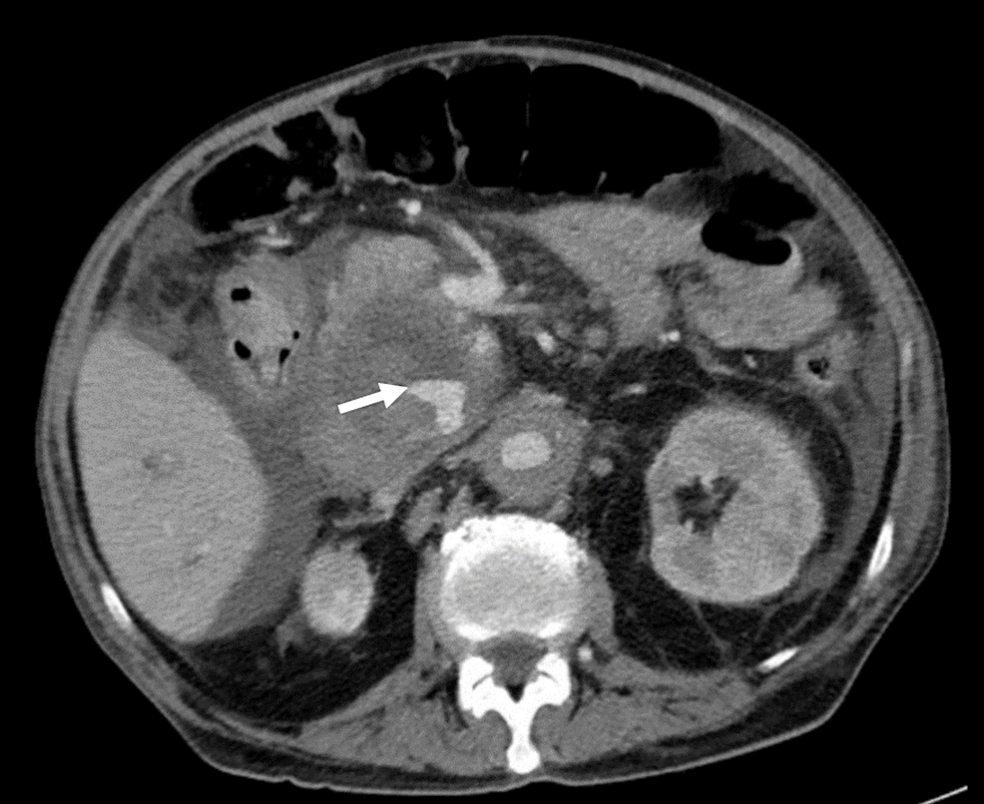
CT with angiogram of abdomen and pelvis (axial view). The white arrow points at a fluid level within the previously described mass represents active bleeding into a hematoma. The contrast enhancement of the fluid level suggests this is an active bleed.

**Figure 3:**
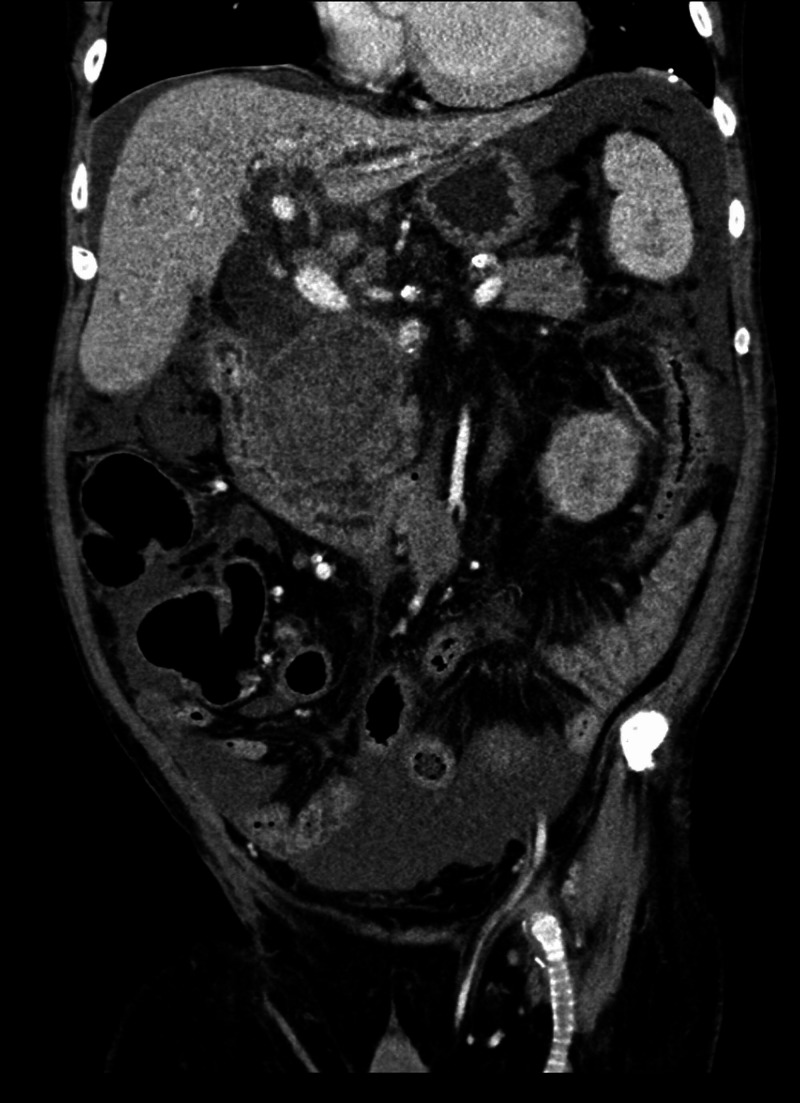
CT with angiogram of abdomen and pelvis (coronal view). The coronal view of the same CT angiogram as Figure 2. The large hematoma is again visualized. Within the liver, intrahepatic biliary dilatation can be appreciated.

Based on this presentation and findings, the patient was admitted to the hospital. Given that the large heterogeneous structure seen on the non-contrast CT was close to the SMA and the head of the pancreas, the differential diagnosis included ruptured SMA aneurysm, SMA pseudoaneurysm, hemorrhagic pancreatic mass, and pancreatic cancer. The modality and urgency of treatment of each of these diagnoses vary greatly. Despite the patient’s acute kidney injury, a CT angiography of the abdomen was performed (Figures 2, 3) as a large SMA aneurysm or pseudoaneurysm would potentially necessitate urgent interventions by either vascular surgery or interventional radiology.

The CT angiography of the abdomen revealed fusiform aneurysmal degeneration of the proximal abdominal aorta. There was an overall beaded appearance, irregular narrowing, and mural thickening involving multiple visceral media and large size arteries, including the celiac artery, splenic artery, superior mesenteric artery, gastroduodenal artery, and right renal artery. A replaced right hepatic artery arising off the SMA extended into the previously described large heterogeneous mass, which appeared to be a hematoma 9.1 x 6.8 x 8.4 cm in size, centered posterior to the head of the pancreas. There were multiple areas of contained active bleeding and pseudoaneurysm formation within this hematoma marginating from branches of the replaced right hepatic artery (Figures 2, 3). Given these concerning findings, vascular surgery and interventional radiology were urgently consulted, but the degree of involvement of multiple vessels precluded any interventions. The patient was discharged with home hospice and passed away two weeks later.

## Discussion

VAPAs are rare vascular lesions that occur when a tear develops in the vessel wall of a visceral artery and a periarterial hematoma forms. The underlying cause may be related to atherosclerosis, trauma, iatrogenic injury, infection, inflammatory states, vasculitis, and connective tissue disorders such as Ehlers-Danlos syndrome. The most common presentation includes symptoms of abdominal pain and anemia. This case was particularly unusual with multiple VAPAs bleeding into a large hematoma causing extrinsic biliary compression, jaundice, and acute pancreatitis. To our knowledge, this has only been reported in two prior case reports [4,5]. While acute pancreatitis is known to occasionally cause pancreatic pseudoaneurysm, this case describes the far more unusual situation of a pseudoaneurysm causing acute pancreatitis. This case also represented a diagnostic challenge in that making the diagnosis necessitated a contrasted CT scan despite the patient having severe acute kidney injury. The renal failure was likely at least partially due to compromise of the right renal artery from the same process that was causing the hepatic artery VAPA.

VAPAs can have life-threatening consequences and should be diagnosed promptly. For example, in our case the leading differential diagnosis was gallstone pancreatitis before appropriate cross-sectional imaging was obtained. The risk of rupture with VAPAs has been reported to be as high as 76.3%, leading to massive intra-abdominal hemorrhage with mortality rates ranging from 25% to 70% [1,2]. Due to this high risk of rupture, treatment of VAPAs is considered urgent. Interventional radiology or vascular surgery are typically involved in the treatment, with options including arterial bypass surgery, vessel ligature, embolization, or endovascular stent placement [6,7]. 

## Conclusions

Clinicians need to recognize that visceral artery pseudoaneurysms require urgent treatment as they can be fatal. The underlying etiology and clinical presentation of VAPAs are quite diverse, which makes diagnosis challenging without an imaging study that reveals the VAPA. The presence of risk factors such as a recent vascular procedure or prior history of vasculopathy may offer some clues to assist in diagnosis. While this case describes one particularly unusual presentation of VAPA involving acute pancreatitis, obstructive jaundice, and acute kidney injury, it also highlights how the size and location of a VAPA dictate the clinical presentation.
